# Inhaled corticosteroids downregulate the SARS-CoV-2 receptor ACE2 in COPD through suppression of type I interferon

**DOI:** 10.1016/j.jaci.2020.09.034

**Published:** 2021-02

**Authors:** Lydia J. Finney, Nicholas Glanville, Hugo Farne, Julia Aniscenko, Peter Fenwick, Samuel V. Kemp, Maria-Belen Trujillo-Torralbo, Su Ling Loo, Maria Adelaide Calderazzo, Jadwiga A. Wedzicha, Patrick Mallia, Nathan W. Bartlett, Sebastian L. Johnston, Aran Singanayagam

**Affiliations:** a National Heart and Lung Institute, Imperial College London, London, United Kingdom; b Prokarium, London BioScience Innovation Centre, London, United Kingdom; c Royal Brompton and Harefield NHS Trust, London, United Kingdom; d Faculty of Health and Medicine and Priority Research Centre for Healthy Lungs, Hunter Medical Research Institute and University of Newcastle, Newcastle, United Kingdom

**Keywords:** COPD, COVID-19, inhaled corticosteroids, viral infection, ACE2, Angiotensin-converting enzyme-2, BEC, Bronchial epithelial cell, BSG, Basigin, COPD, Chronic obstructive pulmonary disease, COVID-19, Coronavirus disease 2019, FP, Fluticasone propionate, GR, Glucocorticoid receptor, ICS, Inhaled corticosteroid, ISG, Interferon-stimulated gene, TMPRSS2, Transmembrane serine protease 2

## Abstract

**Background:**

The mechanisms underlying altered susceptibility and propensity to severe Coronavirus disease 2019 (COVID-19) disease in at-risk groups such as patients with chronic obstructive pulmonary disease (COPD) are poorly understood. Inhaled corticosteroids (ICSs) are widely used in COPD, but the extent to which these therapies protect or expose patients to risk of severe COVID-19 is unknown.

**Objective:**

The aim of this study was to evaluate the effect of ICSs following pulmonary expression of the SARS-CoV-2 viral entry receptor angiotensin-converting enzyme-2 (ACE2).

**Methods:**

We evaluated the effect of ICS administration on pulmonary ACE2 expression *in vitro* in human airway epithelial cell cultures and *in vivo* in mouse models of ICS administration. Mice deficient in the type I IFN-α/β receptor (*Ifnar1*^−/−^) and administration of exogenous IFN-β were used to study the functional role of type-I interferon signaling in ACE2 expression. We compared sputum ACE2 expression in patients with COPD stratified according to use or nonuse of ICS.

**Results:**

ICS administration attenuated ACE2 expression in mice, an effect that was reversed by exogenous IFN-β administration, and *Ifnar1*^−/−^ mice had reduced ACE2 expression, indicating that type I interferon contributes mechanistically to this effect. ICS administration attenuated expression of *ACE2* in airway epithelial cell cultures from patients with COPD and in mice with elastase-induced COPD-like changes. Compared with ICS nonusers, patients with COPD who were taking ICSs also had reduced sputum expression of *ACE2*.

**Conclusion:**

ICS therapies in COPD reduce expression of the SARS-CoV-2 entry receptor ACE2. This effect may thus contribute to altered susceptibility to COVID-19 in patients with COPD.

## Graphical abstract

**Figure:**
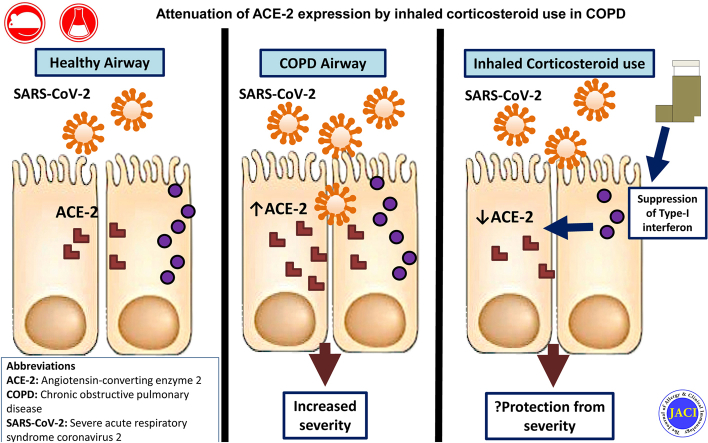

Coronavirus disease 2019 (COVID-19) caused by SARS-CoV-2 infection is a new rapidly spreading infection that can cause a spectrum of disease, ranging from a mild self-limiting upper respiratory tract illness to severe respiratory failure requiring ventilatory support. Current guidance advocates that high-risk individuals, including those with chronic lung diseases such as severe asthma and COPD, should be shielded to reduce risk of infection with SARS-CoV-2.^1^ This guidance is based on extensive prior knowledge that these conditions are exquisitely susceptible to being exacerbated by a range of respiratory virus infections.^2^ However, early evidence has indicated that the prevalence of COPD among hospitalized individuals with COVID-19 may be lower than in the general population, in contrast to the prevalence of other chronic comorbidities such as hypertension and diabetes, raising speculation of a possible protective phenotype.^3^ Conversely, COPD has been shown to be associated with greater risk of COVID-19–related mortality, and severe asthma may perhaps also be associated increased risk, with 1 report suggesting this possibility.^4^^,^^5^ Therefore, these patients could theoretically be protected from acquiring the infection but paradoxically at increased risk of complications if they become infected.

Inhaled corticosteroids (ICSs) are mainstay therapies for airway diseases, and they confer beneficial effects including protection against exacerbations,^6^^,^^7^ suggesting that these drugs may reduce the risk of virus acquisition or alternatively suppress virus-induced inflammation and prevent symptomatic manifestations. Conversely, we and others have shown that ICSs have the adverse effect of suppressing innate immune responses to rhinovirus and influenza infection, leading to increased virus replication,^8^, ^9^, ^10^ although the opposite (protective) effect of ICSs has been reported *in vitro* for the seasonal coronavirus 229E^11^ and SARS-CoV-2.^12^ It is thus unclear whether, overall, ICSs impart a protective or detrimental effect on immune responses to SARS-CoV-2, and the extent to which these widely used drugs protect or expose patients with COPD to COVID-19 is unknown.

SARS-CoV-2 utilizes the entry receptor angiotensin-converting enzyme-2 (ACE2), with priming of the serine protease transmembrane serine protease 2 (TMPRSS2) to gain entry into the respiratory mucosa and cause active infection.^13^ Increased epithelial ACE2 expression has been recently reported in smokers and subjects with COPD^14^ and is postulated to be a factor predisposing these individuals to adverse outcome from COVID-19. Conversely, ACE2 is downregulated in asthma,^15^ an effect that may be due to the suppressive effects of type 2 cytokines^16^ or related to ICS use.^17^ Emerging evidence also indicates that ACE2 expression colocalizes with immune genes involved in interferon signaling pathways.^18^ Moreover, Ziegler et al recently elucidated that *ACE2* is an interferon-stimulated gene (ISG) in human respiratory epithelial cells,^19^ indicating that antiviral pathways may be important in regulation of pulmonary ACE2 expression. We have previously reported that ICS potently suppress epithelial expression of type I interferons and ISGs in a range of *in vitro* and *in vivo* COPD models,^8^ and it is plausible that ICS-mediated suppression of interferon might drive downregulation of ACE2 in the lungs and thus be an important determinant of susceptibility to SARS-CoV-2 in patients with chronic lung disease.

Here, we show that ICS administration attenuates pulmonary expression of ACE2, an effect observed consistently across a range of human and animal COPD models. We demonstrate that the downregulation of ACE2 is mechanistically related to suppression of type I interferon by ICS. These data indicate that use of ICS therapies alters expression of the SARS-CoV-2 entry receptor and may thus contribute to altered susceptibility to COVID-19 in patients with COPD.

## Methods

### The St Mary’s Hospital COPD cohort

A cohort of 40 individuals previously recruited for a longitudinal study carried out at St Mary’s hospital between June 2011 and December 2013 was used to investigate the relationship between ICS use and ACE2 expression. The study received ethical approval from the East London Research Ethics Committee (study no. 11/LL/0229). All included subjects had spirometry-confirmed COPD and were seen when clinically stable (no episodes of acute infection, antibiotic treatment, or oral corticosteroid treatment within the previous 8 weeks). All patients underwent clinical assessment and spirometry and had spontaneous or induced sputum taken and processed as previously described.^20^^,^^21^ Subjects were stratified on the basis of current use or nonuse of ICSs, either in a single-agent inhaler or in combination with bronchodilators.

### Treatment of cultured BECs

Primary bronchial epithelial cells (BECs) were obtained by bronchoscopy from 6 subjects with spirometry-confirmed COPD and 6 healthy nonsmoking control subjects. The study was approved by the Bromley ethics committee (record 15/LO/1241). Primary cells were cultured in collagen-coated T75 flasks in LH-9 medium until 80% confluent before being seeded at 2.5 × 10^5^ cells per well in a 24-well plate. Cells were treated with 10 nM fluticasone propionate (FP) (Sigma-Aldrich) or vehicle dimethyl sulfoxide, as previously reported.^22^

### Mouse models

Female C57BL/6 mice aged 8 to 10 weeks were used for all studies. The mice were purchased from Charles River Laboratories UK and housed in individually ventilated cages within specific pathogen–free conditions.

*Ifnar*^-−/−^ mice were bred in house on a C57BL/6 background. All animal experiments were carried out under the authority of the UK Home Office within the Animals (Scientific Procedures) Act 1986 (project licence no 70/7234).

FP, budesonide, or beclomethasone powder (Sigma-Aldrich, Gillingham, United Kingdom) was resuspended in dimethyl sulfoxide at a concentration of 357 μg/mL and then diluted 1:1000 in sterile PBS. The mice were treated intranasally under light isofluorane anesthesia with a 50-μL solution containing 20, 6.7, or 2 μg of fluticasone or 20 μg of budesonide or beclomethasone.^8^^,^^23^ The mice were culled for end point analyses at 8, 24, 48, or 96 hours after administration of FP. In some experiments, 2 hours after FP administration, mice were additionally treated intranasally with 50 μL of PBS containing 10^4^ units of recombinant IFN-β (Bio-techne, Abingdon, United Kingdom).^8^

### RNA extraction, cDNA synthesis and quantitative PCR

RNA was extracted from cell lysates of primary airway epithelial cells, human sputum cells, or mouse lung tissue by using an RNeasy kit (Qiagen, Manchester, United Kingdom); 2 μg was utilized for cDNA synthesis by using the Omniscript RT kit (Qiagen, Manchester, United Kingdom). Quantitative PCR was carried out by using previously described specific primers and probes and normalized to the 18S rRNA housekeeping gene.^8^ Data were obtained from 2 technical replicates and expressed as fold change in ΔΔ*C*_T_ from the respective control groups. Reactions were analyzed by using the ABI 7500 real-time PCR machine (Applied Biosystems, Warrington, United Kingdom).

### Protein assays

A commercially available ELISA DuoSet kit (Abcam, Cambridge, United Kingdom) was used to measure total ACE2 concentrations in mouse lung homogenate. The lower limit of detection for this assay is 10 pg/mL. IFN-λ concentrations in mouse bronchoalveolar lavage were also measured by using a commercially available ELISA DuoSet kit (Bio-techne, Abingdon, United Kingdom), as previously reported.^8^

### Statistical analyses

For animal experiments, group sizes of 5 mice per condition were used; the data are presented as means plus or minus SEMs representative of at least 2 independent experiments. The data were analyzed by 1-way ANOVA with the Bonferroni multiple comparison test. mRNA expression in sputum cells of ICS users versus in nonusers or in cell lysates of *ex vivo*–cultured epithelial cells treated with FP was compared by using the Mann-Whitney *U* test. All analyses were performed by using GraphPad Prism software, version 8 (GraphPad Software, La Jolla, Calif). Differences were considered statistically significant when *P* was less than .05.

## Results

### Pulmonary *ACE2* mRNA expression is reduced in patients with COPD who are taking ICSs

**Fig 1:**
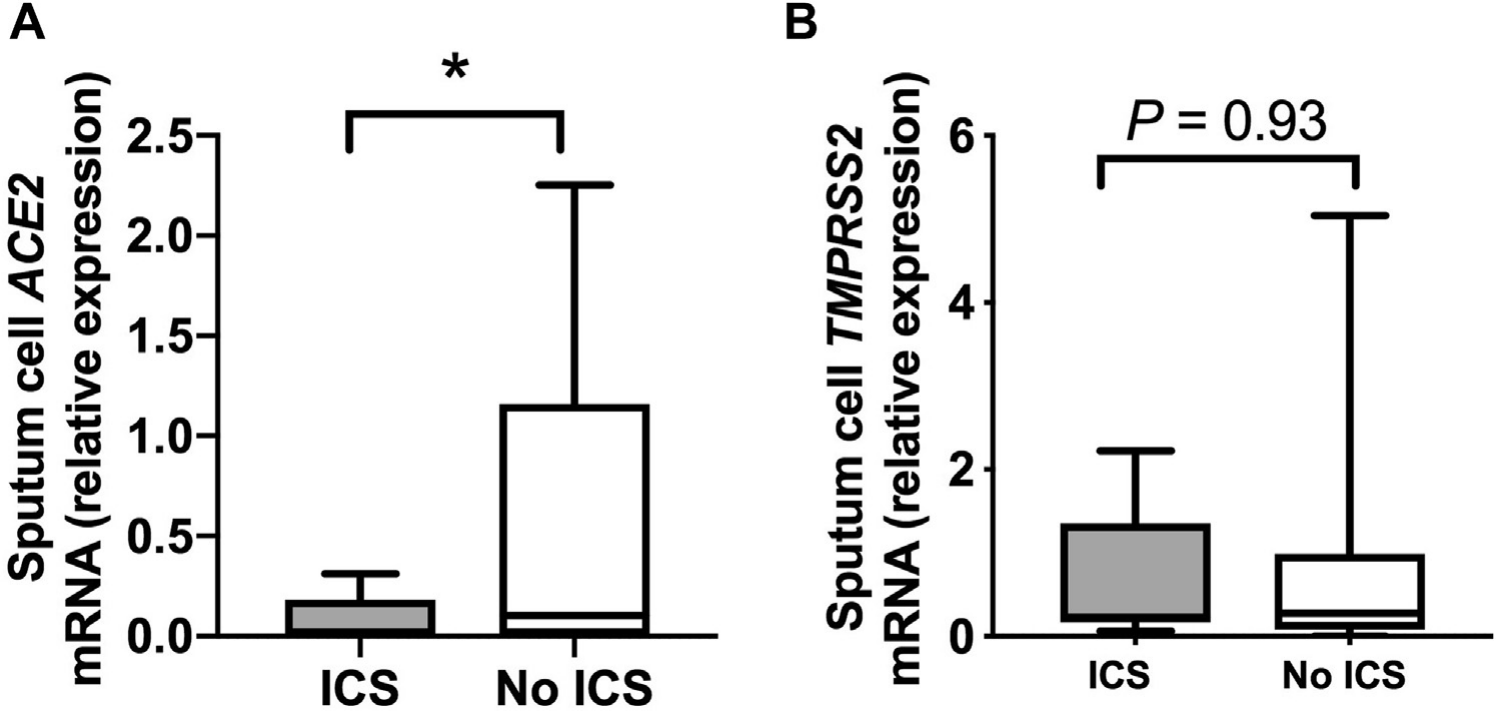
Sputum gene expression of *ACE2* and *TMPRSS2* in subjects with COPD stratified according to use or nonuse of ICSs. Sputum samples were taken from a cohort of patients with COPD when clinically stable for at least 6 weeks. Patients were stratified according to current use or nonuse of ICSs (n = 18 per group). Sputum cell mRNA expression of *ACE2* (**A**) and *TMPRSS2* (**B**) was measured by quantitative PCR. Box and whisker plots show median (*line within box*), interquartile range (*box*) and minimum to maximum (*whiskers*). Statistical comparisons made by using the Mann-Whitney *U* test. ∗*P* < .05.

**Fig E1:**
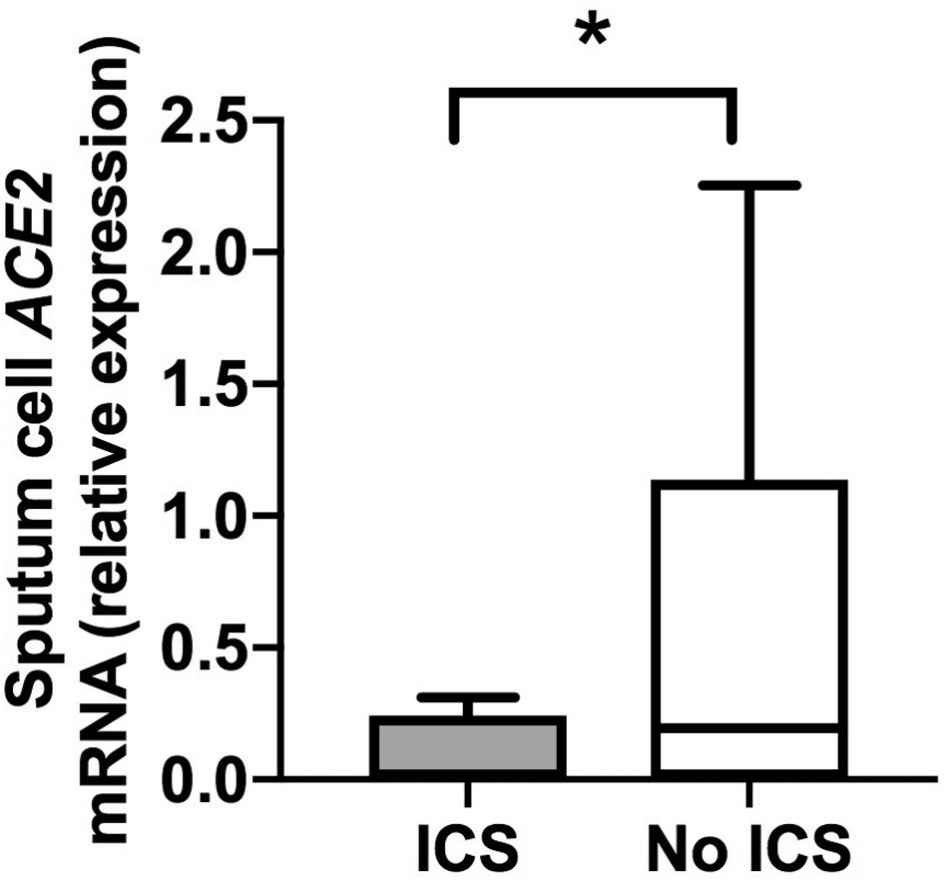
Sputum gene expression of *ACE2* in subjects with COPD stratified according to use or nonuse of ICSs, excluding subjects with bronchodilator reversibility. Sputum samples were taken from a cohort of patients with COPD when clinically stable for at least 6 weeks. Patients were stratified according to current use or nonuse of ICSs (n = 17 ICS users and 15 ICS nonusers). Sputum cell mRNA expression of *ACE2* was measured by quantitative PCR. Box and whisker plots show median (*line within box*), interquartile range (*box*), and minimum to maximum (*whiskers*). Statistical comparisons made by using the Mann-Whitney *U* test. ∗*P* < .05.

**Fig E2:**
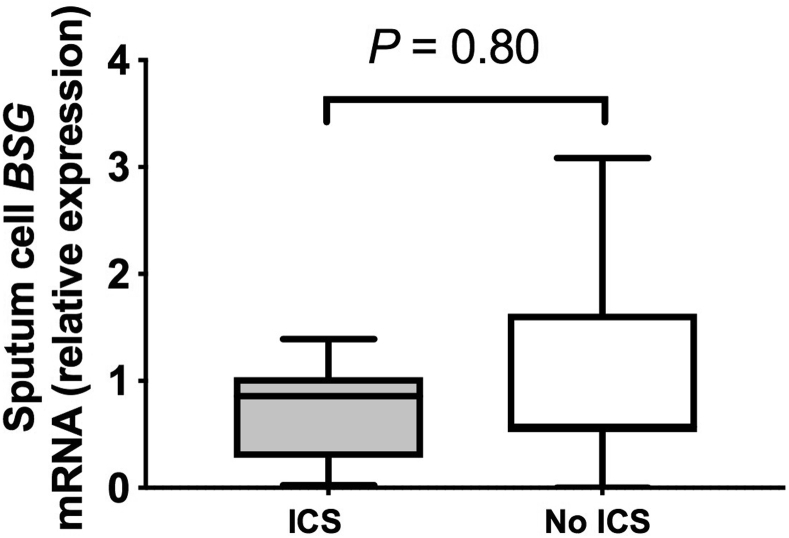
Sputum gene expression of *BSG* in subjects with COPD stratified according to ICS use. Sputum samples were taken from a cohort of patients with COPD when clinically stable for at least 6 weeks. Patients were stratified according to current use or nonuse of ICSs (n = 18 per group). Sputum cell mRNA expression of *BSG* was measured by quantitative PCR. Box and whisker plots show median (*line within box*), interquartile range (*box*), and minimum to maximum (*whiskers*). Statistical comparisons made by using the Mann-Whitney *U* test.

Recent data indicate that sputum expression of *ACE2* mRNA is reduced in subjects with asthma who are taking ICSs,^17^ but whether similar suppression occurs in the context of COPD is unclear. We therefore initially used a community-based cohort of 40 subjects with COPD^20^ to determine whether ICS use affects ACE2 expression in individuals with COPD. Of the 40 subjects, 36 had sufficient sample for evaluation and were stratified according to current use (n = 18) or nonuse (n = 18) of ICSs. There were no significant differences between these groups in terms of age, disease severity, smoking status, or other comorbidities that are known to affect ACE2 expression and/or be associated with increased risk of COVID-19 (Table I). Sputum cell *ACE2* mRNA expression was detectable in 22 of 36 subjects with COPD (61.1%), and consistent with prior observations in subjects with asthma,^17^ it was significantly reduced in ICS users compared with in nonusers (Fig 1, *A*). This effect was consistently observed in a subgroup analysis following exclusion of patients with significant bronchodilator reversibility (>12%) (see Fig E1 in this article's Online Repository at www.jacionline.org). Sputum cell expression of the serine protease TMPRSS2, which is used by SARS-CoV-2 for mucosal entry,^24^ was detectable in all subjects, with no significant difference observed between ICS users and nonusers (Fig 1, *B*). Similarly, the alternative SARS-CoV receptor CD147^25^ (the gene basigin [*BSG*]) was detectable in all subjects, with no difference observed between ICS users and nonusers (see Fig E2 in this article's Online Repository at www.jacionline.org).

**Table I:** Demographic and clinical characteristics of patients with COPD included in the mRNA analyses in Fig 1 and Fig E1

Characteristic	ICS users (n = 18)	ICS nonusers (n = 18)	*P* value
Age (y), mean (range)	68.5 (61.25-73.5)	67 (62.25-70.75)	.61
Male, no. (%)	12 (66.7%)	14 (77.8%)	.71
FEV_1_ (% predicted), mean (range)	65 (50-77)	67 (63-82)	.43
GOLD stage I/II/III/IV (no.)	3/11/2/2	5/10/3/0	**—**
Eosinophils >300 cells/μL, no. (%)	5 (27.8%)	0 (0%)	.046
Prior exacerbations in preceding year (no.), mean (range)	1.5 (1-4)	1 (0-1)	
Current smoker, no. (%)	6 (33.3%)	7 (38.9%)	1.0
Comorbidities, no. (%)			
Diabetes	1 (5.6%)	2 (11.1%)	1.0
Hypertension	2 (11.1%)	0 (0%)	.49
Obesity	3 (16.7%)	2 (11.1%)	1.0
Ischemic heart disease	3 (16.7%)	2 (11.1%)	1.0
ICS type, no. (%)			
Fluticasone	8 (44.4%)	n/a	**—**
Budesonide	9 (50%)		
Beclomethasone	1 (5.6%)		

*n/a*, Not applicable.

### ICS administration suppresses pulmonary expression of ACE2 in mice

**Fig 2:**
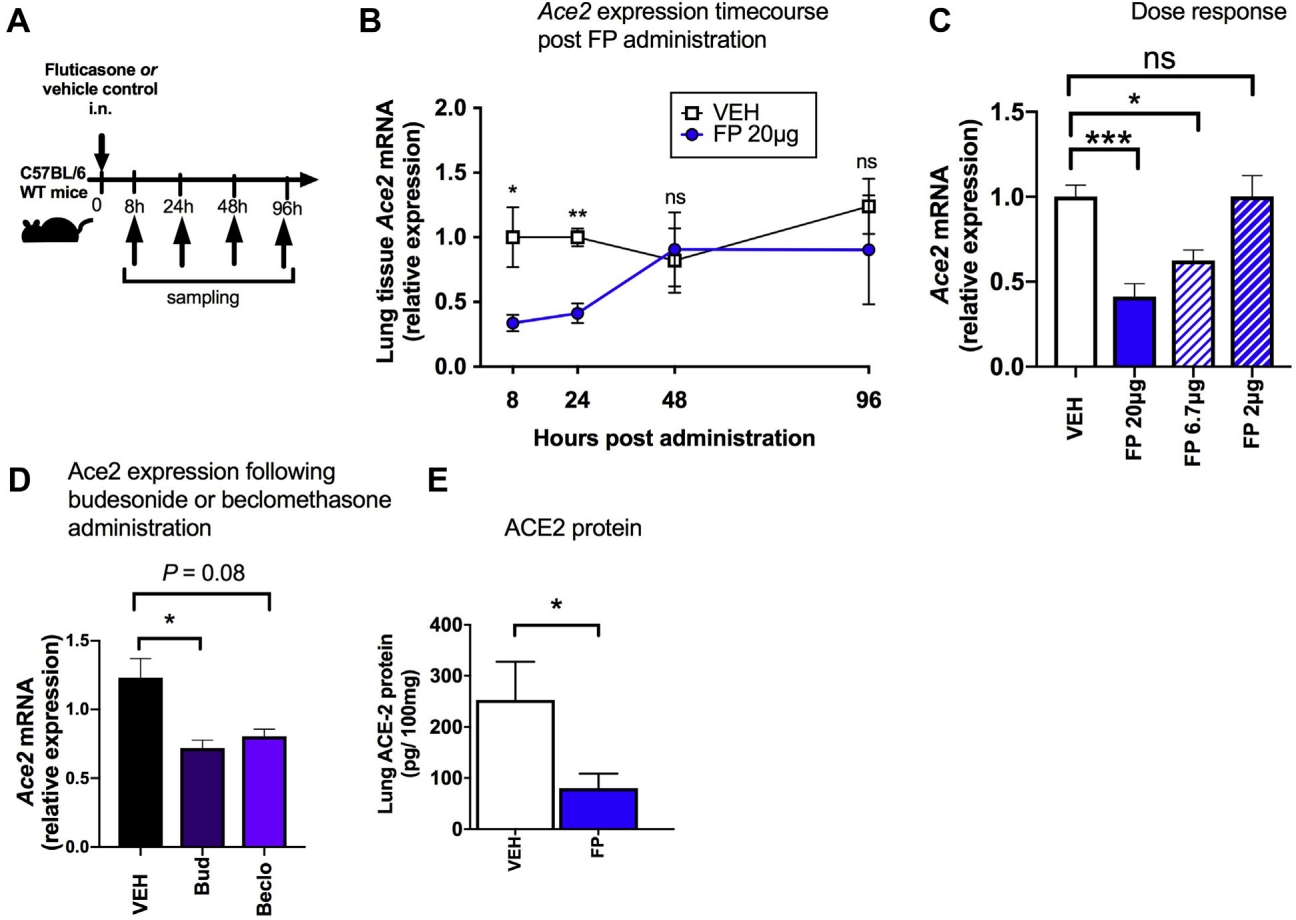
ICS administration downregulates ACE2 expression in mouse lung. **A,** C57BL/6 mice were treated intranasally (i.n.) with a single dose of FP or vehicle (VEH) dimethyl sulfoxide control. **B,** Lung *Ace2* mRNA expression was measured by quantitative PCR (qPCR) at the indicated time points following administration of 20 μg of FP. **C,** Lung *Ace2* mRNA expression was measured by qPCR at 24 hours following single-dose administration of FP at doses of 20, 6.7, and 2 μg. **D,** Lung *Ace2* mRNA expression was measured by qPCR at 24 hours following single-dose administration of 20 μg of budesonide (Bud), beclomethasone (Beclo), or VEH control. **E,** ACE2 protein in lung tissue homogenate measured by ELISA at 24 hours following single-dose administration of 20 μg of FP. Data are shown as means ± SEMs of 4 or 5 mice per treatment group from a single experiment, representative of at least 2 independent experiments. Data were analyzed by using the Mann-Whitney *U* test or 1-way ANOVA with Bonferroni posttest. ∗*P* < .05; ∗∗∗*P* < .001. *ns*, Nonsignificant.

**Fig E3:**
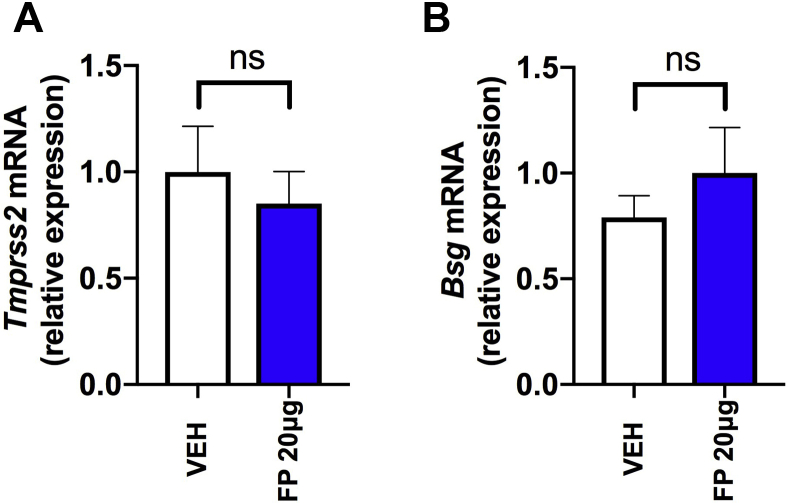
No effect of FP administration on expression of *Tmprss2* or *Bsg* in mouse lung. C57BL/6 mice were treated intranasally with a single 20-μg dose of FP or vehicle dimethyl sulfoxide control. Lung expression of *Tmprss2* (**A**) and lung *Bsg* (**B**) mRNA was measured by quantitative PCR at 8 hours following FP administration. Data are from the same experiment as shown in Fig 2 and represent means ± SEMs of 5 mice per treatment group from a single experiment that is representative of at least 2 independent experiments. Data were analyzed by 1-way ANOVA with Bonferroni posttest. *ns*, Nonsignificant.

Given that cause and effect cannot be inferred from a cross-sectional human study, we next evaluated whether experimental pulmonary administration of the ICS FP in mice, at a dose previously shown to induce lung glucocorticoid receptor (GR) activation,^8^^,^^23^ had similar effects on *Ace2* expression. A single administration of 20 μg of FP in mice (Fig 2, *A*) induced significant downregulation of pulmonary *Ace2* mRNA expression at 8 hours (a time point at which we have also previously shown that significant GR activation occurs^8^). This effect persisted at 24 hours after administration but had resolved from 48 hours onward (Fig 2, *B*). Consistent with the effects observed in human sputum, FP administration had no effect on expression of *Tmprss2* or *Bsg* in mouse lung (see Fig E3 in this article's Online Repository at www.jacionline.org). Suppression of *Ace2* by FP occurred in a dose-dependent manner with loss of suppression at a 10-fold lower concentration (2 μg) (Fig 2, *C*), a dose at which effects on GR activation are also lost.^8^ We observed similar suppression of lung *Ace2* mRNA expression with administration of 20 μg of the other commonly used ICSs budesonide and beclomethasone, suggesting that the effect of ICS on *Ace2* is not class dependent (Fig 2, *D*). To corroborate the effects observed on *Ace2* mRNA expression, we subsequently measured protein levels in lung homogenate of ICS-treated mice by ELISA. We observed similar suppression of total lung ACE2 protein occurring at 24 hours after administration, which is consistent with the effects observed at the mRNA level (Fig 2, *E*).

### Downregulation of ACE2 by FP is functionally related to suppression of type I interferon

**Fig 3:**
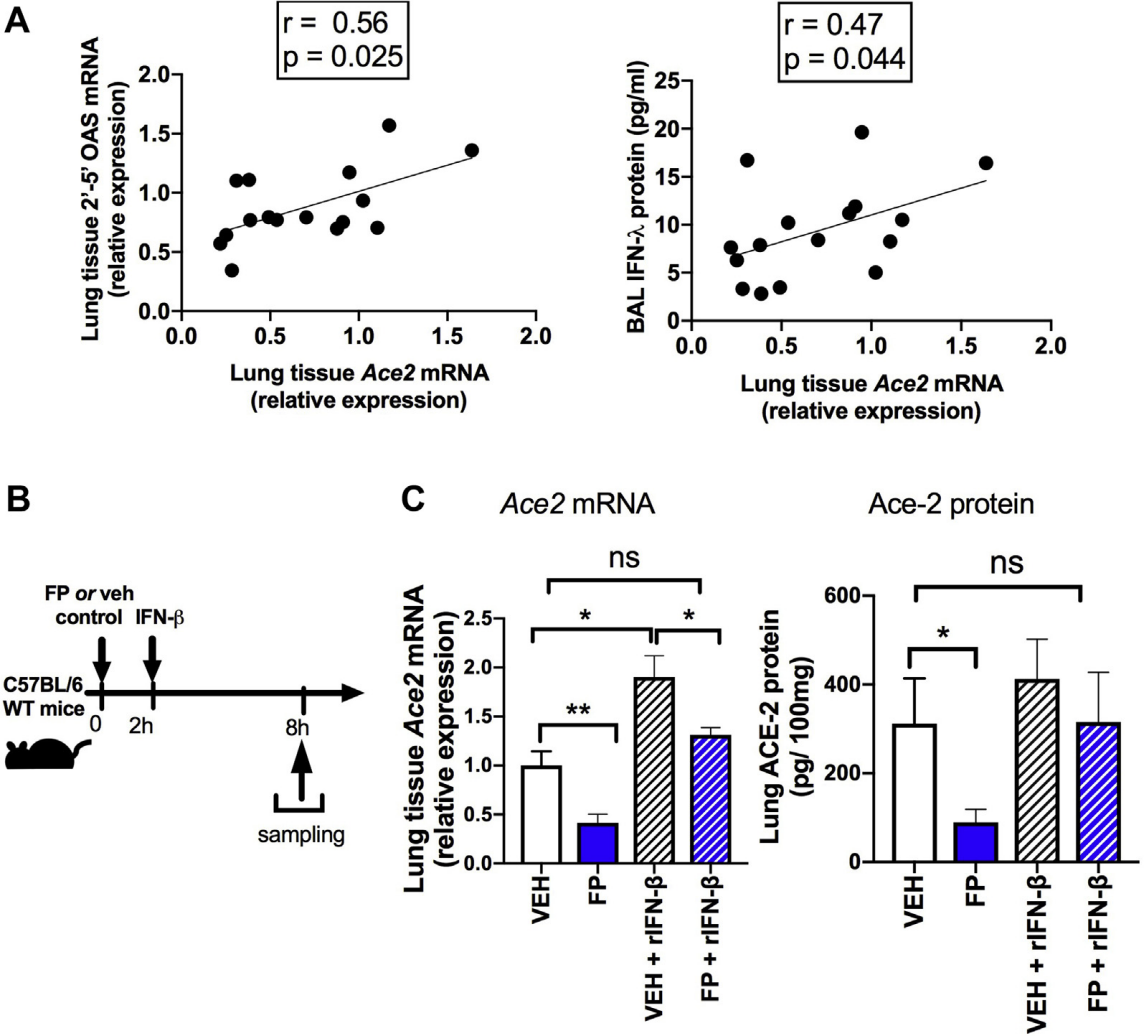
Downregulation of ACE2 by an ICS is functionally related to suppression of type I interferon. **A,** Correlation between lung *Ace2* mRNA and lung 2',5'*-*oligoadenylate synthetase and BAL IFN-λ in C57BL/6 mice. **B,** C57BL/6 mice were treated intranasally with FP (20μg) or vehicle (Veh) dimethyl sulfoxide control and additionally with recombinant IFN-β or PBS control. **C,** Lung *Ace2* mRNA expression and lung ACE2 protein concentrations were measured at 8 hours after FP administration. Data shown as means ± SEMs of 4 or 5 mice per treatment group from a single experiment representative of at least 2 independent experiments. Data were analyzed by using Spearman rank correlation (**A**) or 1-way ANOVA with Bonferroni posttest (**C**). ∗*P* < .05; ∗∗ *P* < .01. *ns,* Nonsignificant.

ACE2 has recently been reported to colocalize with expression of type I interferon–related genes^18^^,^^24^ and has also been shown to be an interferon-stimulated gene (ISG) in the respiratory tract,^26^ suggesting that type I interferon may be a major regulator of pulmonary ACE2 expression. Consistent with this, we observed that basal lung *Ace2* expression in mice positively correlated with type I interferon–inducible mediators, including mRNA expression of 2',5'-oligoadenylate synthetase and bronchoalveolar lavage concentrations of IFN-λ in a combined analysis of FP and vehicle-treated mice (Fig 3, *A*). Given our previous data showing that patients with COPD treated with ICSs have reduced basal airway expression of *IFNβ*,^8^ we hypothesized that downregulation of ACE2 by FP may be functionally related to its suppressive effects on type I interferon signaling. Accordingly, recombinant IFN-β administration (Fig 3, *B*) could reverse FP-mediated suppression of *Ace2* mRNA and ACE2 protein (Fig 3, *C*), indicating that the effect of FP on ACE2 expression is functionally related to the suppressive effects on type I interferon.

### *Ifnar*^−/−^ mice have reduced basal expression of ACE2

**Fig 4:**
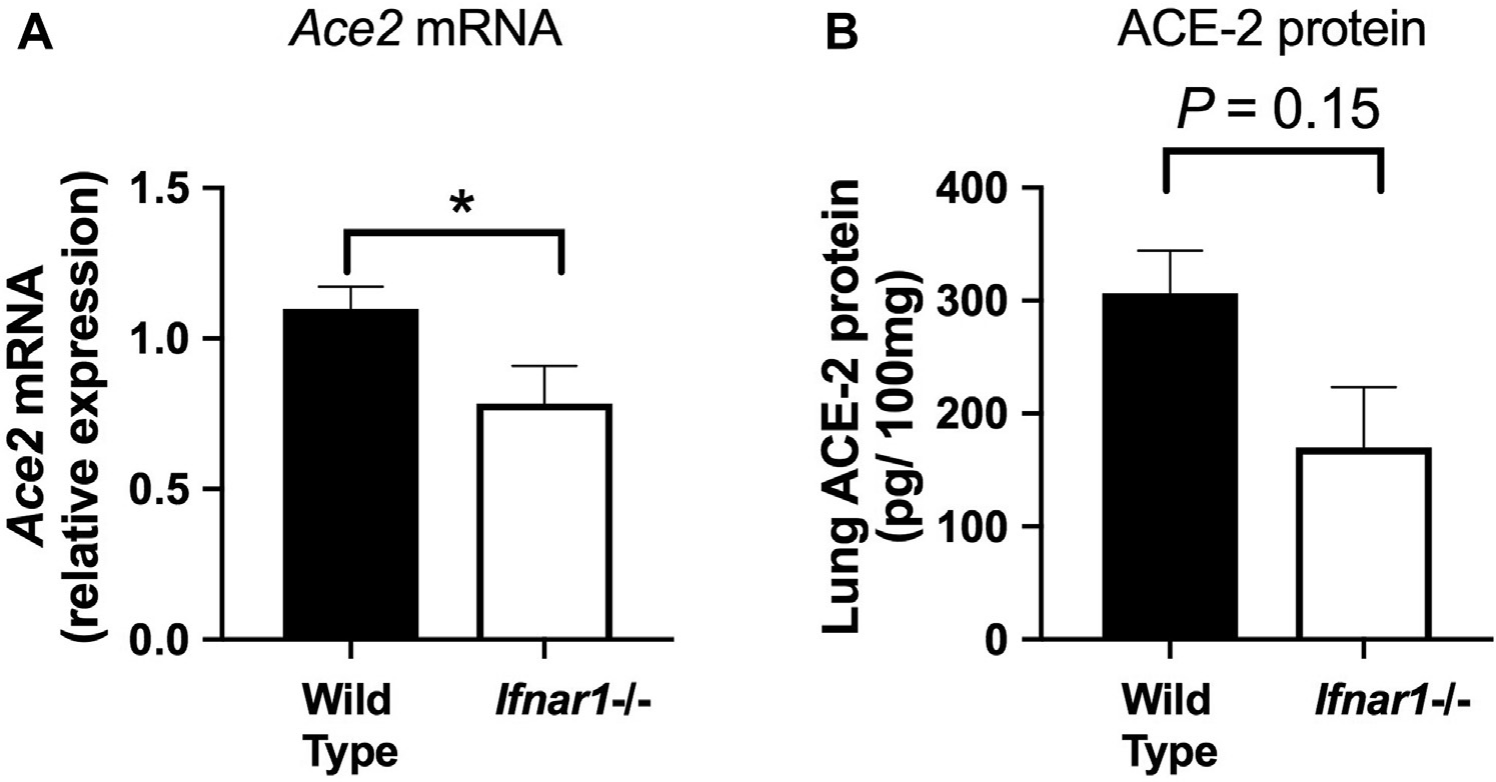
Type I interferon receptor–deficient mice have reduced pulmonary ACE2 expression. Lung tissue was harvested from wild-type or *Ifnar1*^−/−^ C57BL/6 mice. **A,** Lung *Ace2* mRNA expression was measured by quantitative PCR. **B,** Lung ACE2 protein concentration was measured by ELISA. Data are shown as means ± SEMs of 5 mice per treatment group from a single experiment representative of at least 2 independent experiments. Data were analyzed by using a *t* test. ∗*P* < .05.

To further confirm the functional importance of type I interferon in regulating pulmonary ACE2, we evaluated basal pulmonary expression levels in mice deficient in interferon signaling (*Ifnar*^−/−^). Compared with wild-type control mice, the *Ifnar*^−/−^ mice had a small, but statistically significant, reduction in lung *ACE2* mRNA expression (Fig 4, *A*), with a concomitant trend (*P* = .15) toward reduced lung ACE2 protein levels (Fig 4, *B*). These observations further confirm the key regulatory role played by type I interferon signaling in pulmonary expression of ACE2.

### The suppressive effect of ICS following *ACE2* expression occurs at the bronchial epithelium

**Fig 5:**
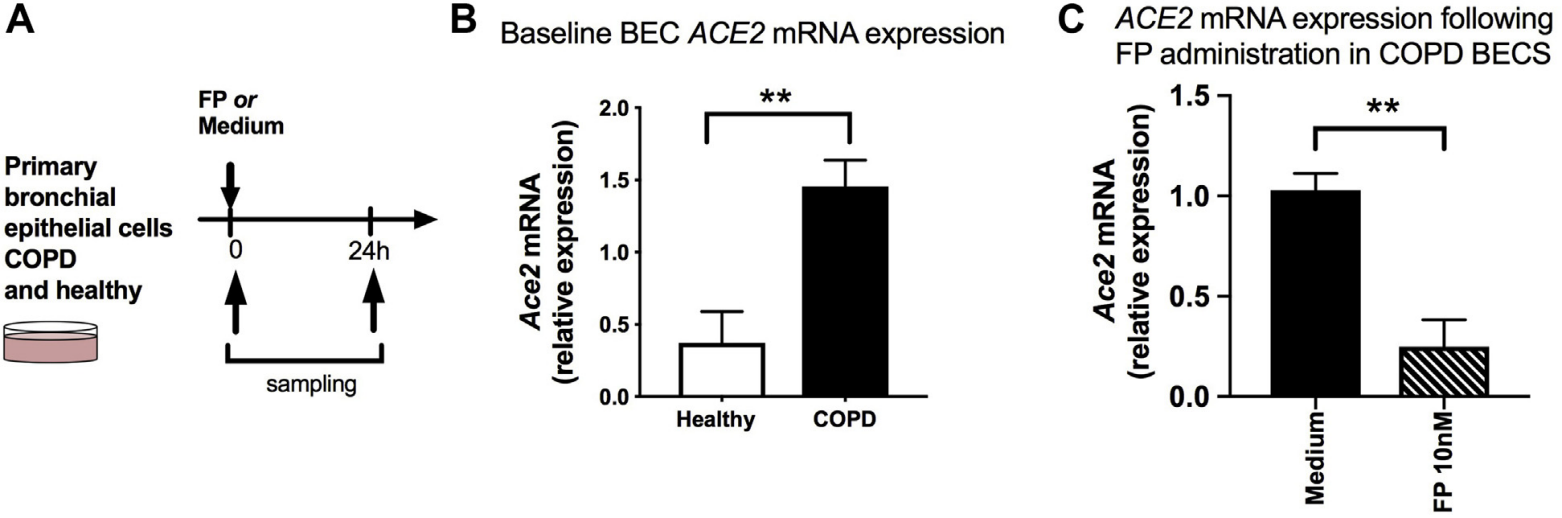
ACE2 expression is increased in COPD and suppressed by fluticasone administration in cultured bronchial epithelial cells (BECs). **A,** Primary BECs from 6 subjects with COPD and 6 healthy control subjects were cultured *ex vivo* and treated with 10 nM FP or medium control. Cell lysates were collected. ACE2 mRNA expression was measured at baseline (**B**) and at 24 hours after FP administration (**C**) in COPD BECs. Data are shown as medians ± interquartile ranges. Statistical comparisons were made by using the Mann-Whitney *U* test. ∗∗*P* < .01.

**Fig E4:**
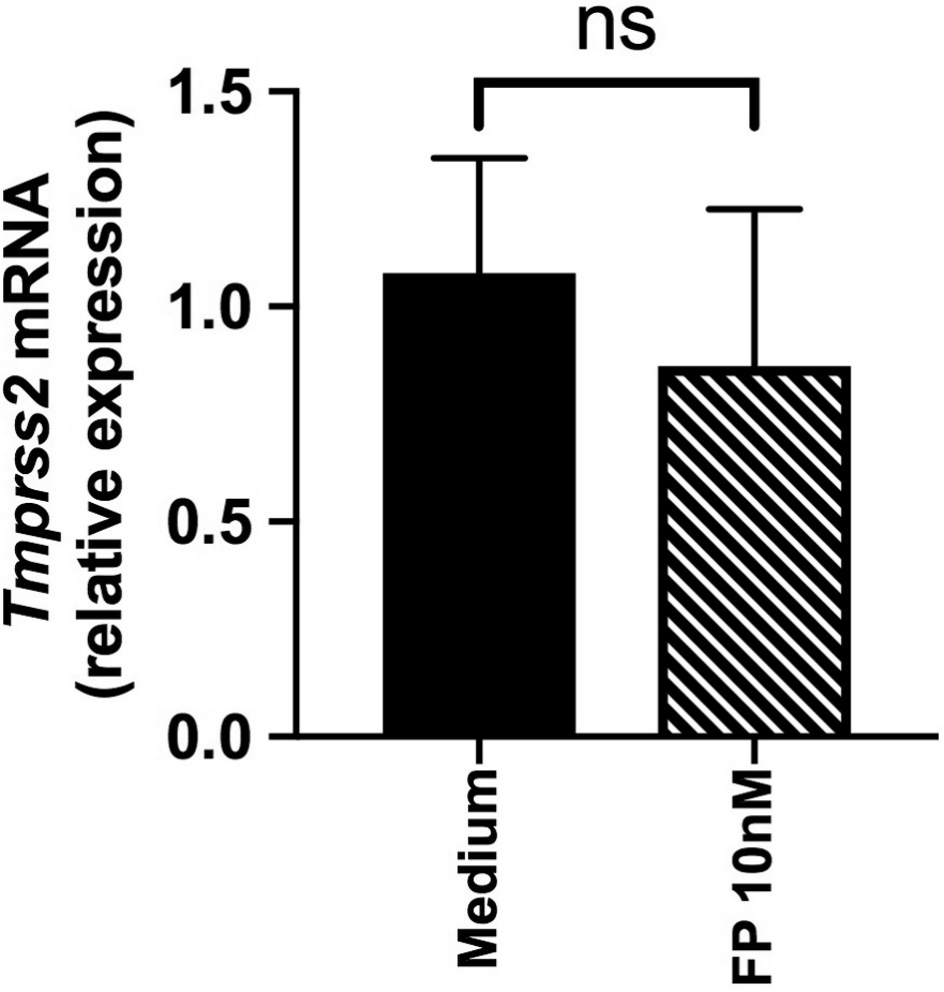
No effect of fluticasone administration on *TMPRSS2* expression in cultured BECs. Primary BECs from 6 subjects with COPD and 6 healthy control subjects were cultured *ex vivo* and treated with 10 nM FP or control medium. Cell lysates were collected. *TMPRSS2* mRNA expression was measured by quantitative PCR. Data are shown as medians ± interquartile range. Statistical comparisons made by using the Mann-Whitney *U* test. *ns*, Nonsignificant.

Existing data indicate that ACE2 is expressed primarily in the nasal and bronchial epithelium and is absent from immune cells.^16^ Given our prior data indicating that FP also exerts its inhibitory effects on immunity principally at the pulmonary epithelium,^8^^,^^22^ we next assessed whether suppressive effects on ACE2 were also observed following *ex vivo* ICS administration in cultured COPD BECs (Fig 5, *A*). The baseline characteristics of the subjects included in these analyses are shown in Table II. In keeping with recent *in situ* expression studies in patients with COPD,^14^ we found that basal expression of *ACE2* was increased by approximately 3-fold in BECs from patients with COPD compared with in healthy nonsmokers (Fig 5, *B*). Consistent with our findings in human ICS users and in the mouse model of ICS administration (Figs 1 and 2), FP administration (at a clinically relevant concentration of 10 nM) induced approximately 75% suppression of *ACE2* expression (Fig 5, *C*). There was no effect of FP administration on *TMPRSS2* expression in COPD BECs (see Fig E4 in this article's Online Repository at www.jacionline.org).

**Table II:** Demographic and clinical variables in subjects with COPD and healthy subjects included in the primary airway epithelial cell experiments (Fig 5)

Variable	Subjects with COPD (n = 6)	Healthy subjects (n = 6)	*P* value
Age (y), mean (range)	70 (67-71.5)	58 (56.5-60.25)	.009
Male, no. (%)	3 (50%)	5 (83.3%)	.54
Current smoker, no. (%)	2 (33.3%)	0 (0%)	.45
Diabetes mellitus, no. (%)	1 (16.7%)	0 (0%)	1.0
Obesity, no. (%)	1 (16.7%)	0 (0%)	1.0
Hypertension, no. (%)	3 (50%)	0 (0%)	.18

### FP downregulates pulmonary ACE2 expression in a mouse model of COPD

**Fig 6:**
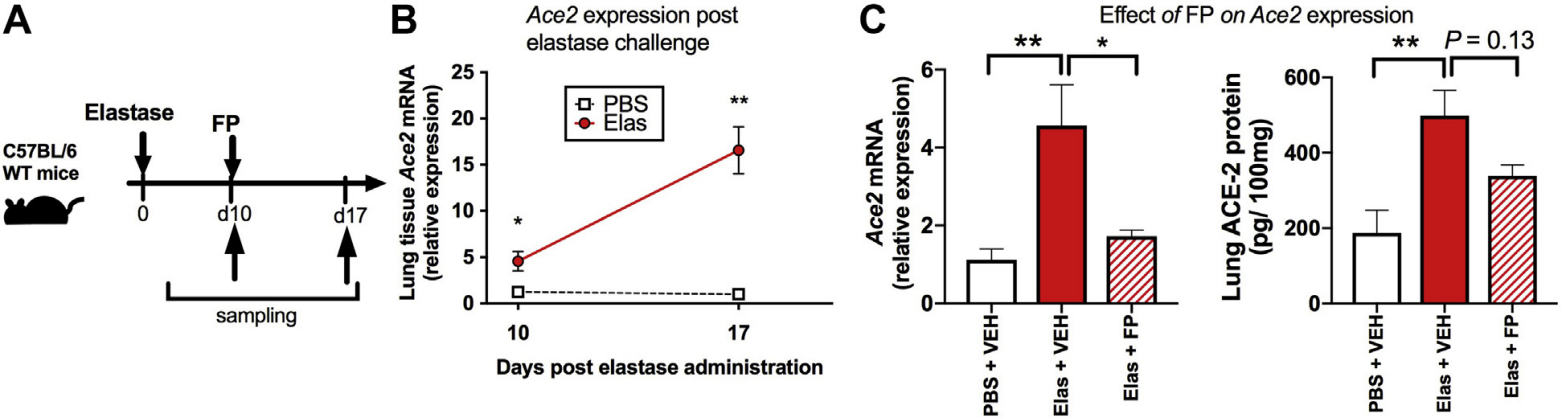
ACE2 expression is increased in a mouse model of COPD and suppressed by fluticasone administration. **A,** C57BL/6 mice were treated intranasally with a single dose of elastase (Elas) or PBS as control and lung tissue harvested at 10 or 17 days later. Some mice were treated with FP or vehicle (VEH) control at 10 days, before sampling 24 hours later. **B,** Lung *Ace2* mRNA expression in Elas- versus PBS-treated mice at 10 and 17 days after treatment. **C,** At 10 days after Elas treatment, C57BL/6 mice were treated with a single dose of 20 μg of FP or VEH control. Lung tissue was harvested at 24 hours after FP administration. *Ace2* mRNA was measured by quantitative PCR (*left panel*), and ACE2 protein was measured by ELISA (*right panel*). Data are shown as means ± SEMs of 5 mice per treatment group from a single experiment. representative of at least 2 independent experiments. Data were analyzed by 1-way ANOVA with Bonferroni posttest. ∗*P* < .05; ∗∗*P* < .01. *ns*, Nonsignificant.

**Fig E5:**
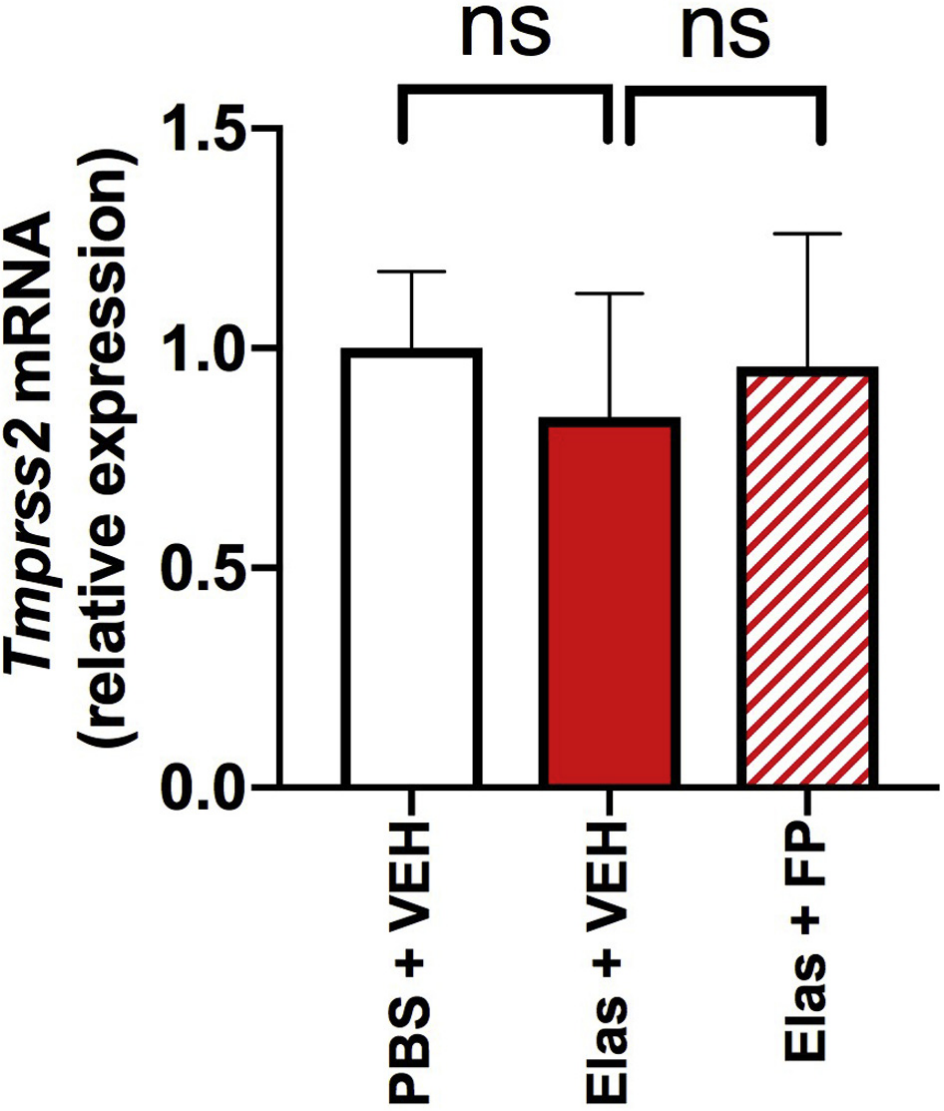
No effect of fluticasone administration on lung *Tmprss*2 expression in a mouse model of COPD. **A,** C57BL/6 were treated intranasally with a single dose of elastase (Elas) or PBS and treated with FP of vehicle (VEH) 10 days later, before sampling 24 hours after. **B,** Lung *Tmprss2* mRNA expression was measured by quantitative PCR. Data shown are means ± SEMs of 5 mice per treatment group from a single experiment representative of at least 2 independent experiments. Data were analyzed by 1-way ANOVA with Bonferroni posttest. *ns*, Nonsignificant.

To further confirm that ICS administration suppresses ACE2 expression in COPD, we used a mouse model of elastase-induced emphysema that recapitulates many hallmark features of the disease in humans.^27^ Mice were treated intranasally with a single dose of porcine pancreatic elastase (Fig 6, *A*); lung ACE2 expression was measured at 10 days after administration (the time point at which COPD-like disease features are established^27^) and a further 7 days later. In keeping with our findings in human COPD cells, elastase-treated mice had significantly increased (∼5-fold) lung *Ace2* expression at 10 days, with further enhancement to more than 15-fold at 17 days (Fig 6, *B*). Administration of a single dose of FP at 10 days attenuated the significant upregulation of lung *Ace2* mRNA (Fig 6, *C*) and ACE2 protein concentrations measured 24 hours later in elastase-treated mice (Fig 6, *D*), but it had no effect on *Tmprss2* (see Fig E5 in this article's Online Repository at www.jacionline.org). Therefore, the suppressive effects of ICS on ACE2 also occur in an *in vivo* model of COPD-like disease.

## Discussion

The mechanisms driving altered susceptibility to COVID-19 in chronic lung diseases, and whether the commonly used ICS therapies promote or protect against infection by SARS-CoV-2 is a crucial question for the field. In this study, we have demonstrated consistently, across a range of human and animal models, that ACE2, a receptor facilitating entry of SARS-CoV-2 into the respiratory tract, is upregulated in COPD and suppressed by ICS treatment. Our studies indicate a novel mechanism for downregulation of ACE2 by ICS through suppression of type I interferon.

There is clear evidence to support the premise that ACE2 mediates cell entry of SARS-CoV2 into the respiratory tract and also acts as a major receptor for SARS-CoV1 and NL63 coronaviruses.^24^^,^^28^^,^^29^ Viral entry occurs as a 2-step process with initial binding of the N-terminal portion of the viral protein to the ACE2 receptor, followed by viral protein cleavage facilitated by the receptor TMPRSS2.^24^ Therapeutic blockade of TMPRSS2 inhibits entry of SARS-CoV-1 and SARS-CoV-2 into cells, supporting a critical role for this protease in viral pathogenesis.^24^ Our data indicate clear downregulation of ACE2 by ICS but with no concomitant effect on TMPRSS2. Although the precise role of ACE2 in the pathogenesis of COVID-19 is not fully characterized, several lines of evidence indicate the important role it plays in mediating virus entry and facilitating disease pathogenesis of SARS-CoVs. In SARS-CoV-1, greater ACE2 expression increases *in vitro* susceptibility to infection.^30^ Moreover, in animal models, overexpression of *Ace2* enhances SARS-CoV-1 entry, anti-ACE2 antibodies can block viral invasion, and reduced pulmonary pathology is observed in *Ace2-*deficient mice.^29^^,^^31^^,^^32^

ACE2 is expressed primarily in the nasal goblet cells and type II pneumocytes within the respiratory tract,^33^ and it is upregulated in subject groups known to be associated with increased disease severity, including elderly individuals^34^ and patients with diabetes,^17^ indicating that it may play a clinically important role in governing susceptibility to virus acquisition and/or development of severe disease in at-risk groups. ACE2 expression has similarly been shown to be increased in COPD: by using combined transcriptomic and immunohistochemical analyses, Leung et al recently demonstrated that epithelial *ACE2* expression is increased in bronchial brushings/tissue samples from subjects with COPD versus in healthy controls,^14^ effects that have also been shown previously in cigarette smoke exposure animal models.^35^ Our data showing increased *ACE2* expression in cultured airway epithelial cells and in an elastase mouse model of COPD are consistent with these findings, although it should be noted that in our *in vitro* experiments, the subjects with COPD were significantly older than the healthy subjects, which may theoretically act as a confounding modulator of *ACE2* expression. The rates of patients with COPD being hospitalized with COVID-19 have been relatively low, ranging from 1.1% in Chinese cohorts to 5% in US cohorts.^36^, ^37^, ^38^, ^39^ However, patients with COPD have greater risk of severe disease and mortality from COVID-19. These data suggest that COPD may be associated with possible protection against the need for hospitalization (possibly because of reduced risk of virus acquisition) but increased propensity to severe disease following infection. The original SARS-CoV-1 pandemic was also characterized by an extremely low prevalence of chronic lung disease comorbidities,^40^^,^^41^ an effect that could also have been driven by ICS-mediated suppression of ACE2 in these subjects. The mechanism underlying these putative alterations in susceptibility has not been extensively explored. Our data suggest that suppression of ACE2 by the commonly used ICS therapies may be 1 important factor that dictates susceptibility in COPD.

Currently, a direct causal link between altered ACE2 expression and increased susceptibility to acquisition of SARS-CoV-2 or subsequent severity has not been proven, and we cannot conclude unequivocally that downregulation of ACE2 by ICSs is an effect that would confer protection clinically. In asthma, a disease in which ICSs are more commonly prescribed than in COPD, *ACE2* expression is reduced compared with that in healthy subjects^15^ and also further attenuated in ICS users.^17^ In contrast to COPD, asthma is less clearly associated with increased COVID-19 mortality,^42^ and the more widespread use of ICS with associated suppression of ACE2 could theoretically be 1 factor driving this. Conversely, it is important to note that there is evidence to suggest that downregulation of ACE2 could also theoretically worsen outcome. In mouse models of experimentally induced acid aspiration and sepsis, genetic deletion of *Ace2* worsens acute lung injury, an effect that is partially rescued by recombinant ACE2 administration.^43^ ACE2 also degrades angiotensin II, which can drive production of proinflammatory cytokines,^44^^,^^45^ which in turn may be detrimental in the context of the hyperinflammation that is characteristic in severe COVID-19.^46^ Downregulation of ACE2 by ICSs could therefore remove critical homeostatic protective functions within the lungs and thereby promote severe disease in COVID-19.

ICS use can also impart a number of other detrimental effects on innate immunity to other respiratory viruses (which may also occur in the context of SARS-CoV-2 infection), including suppression of type I interferon leading to increased virus replication^8^, ^9^, ^10^ and virus-induced pathology, such as mucus hypersecretion and secondary bacterial infection.^8^ We therefore cannot currently ascertain whether the clear suppressive effect of ICS on ACE2 expression that was shown in our experiments would result in ICS use in COPD having overall protective or detrimental effects in the context of clinical disease. Further functional manipulation experiments (eg, SARS-CoV-2 challenge in ICS-treated ACE2-depleted animals), coupled with prospective studies of ICS use as a predictor of susceptibility to SARS-CoV-2 infection, are required to understand the direct implications of this effect. If ACE2 downregulation does confer protection against SARS-CoV-2 infection, then our results would suggest that these therapies should be continued stringently in subjects with asthma and COPD. Ultimately, clinical trials of ICSs in healthy subjects and patients with COPD that examine the outcomes of frequency and/or severity of Sars-CoV-2 infections will be required to determine whether these drugs offer protection against COVID-19. Such trials are ongoing (NCT04416399 and NCT04355637), and results are eagerly awaited. Because ACE2 is also highly expressed in nasal secretory cells, it is also plausible that intranasal steroids used alone or in combination with an ICS could offer further protective benefit. Furthermore, our data also indicate that other therapies currently in trials, such as recombinant IFN-β, could theoretically induce the adverse effect of increasing ACE2 and promoting greater viral entry.

Understanding the mechanism through which ICS suppress expression of ACE2 is important to delineate how this effect could either be harnessed as a protective factor or reversed if deemed to be detrimental. We have previously shown that ICSs can suppress type I interferon both at steady state and during active virus infection.^8^ Here, we have shown that this effect directly drives suppression of ACE2 expression because administration of recombinant IFN-β in combination with FP could reverse the downregulation of ACE2. Furthermore, *Ifnar*^−/−^ mice, which lack type I interferon signaling, also had reduced ACE2, providing additional evidence that type I interferon contributes to this expression. These findings are consistent with recent studies showing that genes relevant to IFN pathways (*IFNAR1* and *IFITM1*) are expressed in *ACE2*^+^ type 2 pneumocytes and that type I interferons can upregulate ACE2 in a range of experimental systems.^24^ We have additionally shown that 2 other commonly used ICSs, budesonide and beclomethasone, have similar effects on ACE2, confirming that, in contrast to impairment of antibacterial immunity,^23^ the suppressive effect of ICS on ACE2 is not class dependent. This is also consistent with the mechanism of suppressed interferon, as we have also shown previously that budesonide suppresses interferon to a degree similar to FP.^8^

In summary, these studies indicate that ICS use in COPD suppresses the expression of the SARS-CoV-2 receptor ACE2 through a type I interferon–dependent mechanism. These effects are likely to contribute to altered susceptibility to COVID-19 in patients to whom these therapies are prescribed. Further studies are now needed to elucidate the precise effects that altered ACE2 expression has on the acquisition of SARS-CoV-2 infection and associated severity in COVID-19.

**Key messages**

• ICS administration attenuates pulmonary expression of ACE2, a key viral entry receptor used by SARS-COV-2.
• This effect may potentially contribute to altered susceptibility to COVID-19 in COPD either beneficially (reduced viral entry) or detrimentally (removal of protective effect of ACE2 against hyperinflammation).
• Clinical trials of ICS in COVID-19 are ongoing and will offer further insight.
